# Detection of Green Asparagus Using Improved Mask R-CNN for Automatic Harvesting

**DOI:** 10.3390/s22239270

**Published:** 2022-11-28

**Authors:** Xiangpeng Liu, Danning Wang, Yani Li, Xiqiang Guan, Chengjin Qin

**Affiliations:** 1 College of Information, Mechanical and Electrical Engineering, Shanghai Normal University, Shanghai 201418, China; 2 School of Engineering and Telecommunications, University of New South Wales, Sydney 2052, Australia; 3 School of Mechanical Engineering, Shanghai Jiao Tong University, Shanghai 200240, China

**Keywords:** agricultural automation, green asparagus detection, DA-Mask RCNN, depth filter, different weather, illumination conditions

## Abstract

Advancements in deep learning and computer vision have led to the discovery of numerous effective solutions to challenging problems in the field of agricultural automation. With the aim to improve the detection precision in the autonomous harvesting process of green asparagus, in this article, we proposed the DA-Mask RCNN model, which utilizes the depth information in the region proposal network. Firstly, the deep residual network and feature pyramid network were combined to form the backbone network. Secondly, the DA-Mask RCNN model added a depth filter to aid the softmax function in anchor classification. Afterwards, the region proposals were further processed by the detection head unit. The training and test images were mainly acquired from different regions in the basin of the Yangtze River. During the capturing process, various weather and illumination conditions were taken into account, including sunny weather, sunny but overshadowed conditions, cloudy weather, and daytime greenhouse conditions as well as nighttime greenhouse conditions. Performance experiments, comparison experiments, and ablation experiments were carried out using the five constructed datasets to verify the effectiveness of the proposed model. Precision, recall, and F1-score values were applied to evaluate the performances of different approaches. The overall experimental results demonstrate that the balance of the precision and speed of the proposed DA-Mask RCNN model outperform those of existing algorithms.

## 1. Introduction

Green asparagus is a variety of asparagus with high nutritional value; it is known as “the king of vegetables” and has been used as human food for thousands of years [1]. To obtain the best taste and nutritional values, green asparagus needs to be harvested in a timely manner after it reaches maturity. In addition, as green asparagus is a high-price seasonal product, the quality of the fresh cut has a significant impact on the price it can reach. The traditional manual harvesting approach is costly, slow, and labor-intensive; therefore, the development of autonomous green asparagus harvesting equipment is of high priority. A form of technology that will realize the effective detection and cutting point acquisition of green asparagus in the field or in the greenhouse is urgently required. Complex lighting in the natural environment [2], mutual occlusion between crops, the interference of weeds, and other factors greatly increase the difficulty of accurately identifying and positioning green asparagus. Moreover, the similarity of appearance of green asparagus stalks and loess in the captured image adds additional complexity to the accuracy of a vision-based green asparagus detection task.

**Figure 1:**
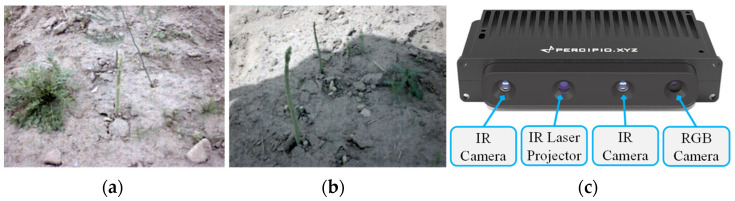
Examples of captured images of green asparagus using Tuyang FM810 Camera: (**a**) Green asparagus in a weedy environment. (**b**) Green asparagus in shade. (**c**) Front view of the camera.

The accuracy and real-time performance of vision-based crop detection in complex agricultural environments are among the significant issues that influence the efficient operation of intelligent agricultural machinery [3]. In this paper, we used green asparagus as our research object, and we developed the depth-aided Mask RCNN model (DA-Mask RCNN), which optimizes the region proposal network (RPN) section of the Mask R-CNN model on the basis of depth information. First of all, the combination of the residual network (ResNet) and the feature pyramid network (FPN) was chosen as the backbone network. The feature extractor sends five feature maps of different dimensions to the next network after extracting features [4], with the aim of improving the processing speed and efficiency of feature extraction. By applying the feature pyramid network, the fusion of shallow features and deep features was achieved. In the meantime, the FPN enhanced the detection accuracy of multi-scale targets by generating various sizes of anchors [4]. Secondly, the depth information was used to optimize the classification of the anchors in the region proposal network, which was originally solely performed by the softmax function. Afterwards, the Distance-IoU (DIoU) loss was employed in the detection head unit as the target regression loss function, which made the position regression faster and more accurate. Finally, the classification, location, and other information acquired by the network were fused with the depth information to obtain the best cutting point of green asparagus. In the experiment, five scenarios with different weather and illumination conditions were considered to verify the performance of the proposed model. Images of green asparagus were captured by a Tuyang FM810 camera, which consists of an RGB camera and two infrared cameras. Examples of the captured images are shown in Figure 1.

## 2. Related Work

At present, most intelligent agricultural machineries used to detect crops are based on machine vision. The fast and accurate detection of crop types, along with positioning, is a prerequisite for the efficient and accurate operation of harvesting machineries [5]. The methods of crop detection and classification are mainly categorized into two types: traditional machine-vision-based approaches [6] and deep-learning-based approaches [7].

Models based on traditional machine vision are comparatively easier to implement and are widely used in many fields of modern agriculture. The predicted point Hough transform based on traditional machine vision was used to fit the navigation route of the greenhouse cucumber picking robot. The use of the gray factor could well segment cucumber stalks and soil, which was 10.25 degrees lower than the average error of the least squares method [8]. Many novel plant vision systems in the context of machine vision technology have been developed to estimate the morphological features of plants, with the aim of determining their growth and health status [9,10,11]. Partial image features were combined with color information to segment apple images using a pixel block segmentation approach in the gray-centered RGB color space [12]. To recognize ripe and unripe pomelo fruit on the trees, Liu et al. [13] used a machine vision algorithm based on an ellipse boundary model to convert images from RGB space to YC_b_C_r_ space. The proposed approach performed better in detecting mature fruit, the color of which is different from green, than in detecting immature green fruit. Traditional machine vision is also widely utilized in multi-classification systems for the identification and localization of fruits and vegetables [14,15,16,17]. Therefore, the accurate position of fruits and vegetables can be obtained in three-dimensional space, and the original monocular vision has been developed to binocular vision or multi-eye vision. In order to achieve automatic weed control, Gai et al. [18] utilized the Kinect v2 sensor to collect field images of broccoli and lettuce. Afterwards, they fused color images and depth images to detect crops at varied growth stages. These methods are mainly based on the combination of one or more elements of crop color, shape, texture, spectrum, and position to identify and position crops. However, in the actual application process, these methods can only be used to detect crops in specific environments. In addition, they are often influenced by factors such as natural illumination, background noise, and the occlusion of branches and leaves, resulting in a reduction in recognition accuracy. In addition, the feature extraction processes within these types of classifiers have certain degrees of human subjectivity, which also influence the robustness of the models [19].

In addition, stereo imaging sensors are increasingly widely used in the field of smart agriculture. The shapes and spatial structures of images can comprehensively show the growth status of crops [20,21,22], which has a competitive advantage in crop detection and classification. A forward-looking light field camera named Lytro LF could explore the process of plant growth and characteristics. It strongly confirmed the assumption that the light field camera proposed above has the potential to be a light-weight and convenient measurement instrument for environmental scene monitoring [23]. Furthermore, an improved RGB camera as well as a kinematic stereo approach can be applied to perform parallax compensation, which provides precise location-related information from plants for lettuce field crop and weed classification [24]. Cui et al. [25] integrated the YOLOv3 model and the DarkNet framework into the matching approach to obtain coordinates in a point cloud image. Meanwhile, they displayed the 3D coordinates’ feature point values in order to classify various target crops. Moreover, it was shown that the measurement of distance between a LiDAR and grapes had sufficient spatial resolution, which can provide high-density 3D point clouds and can be exploited to obtain 3D reconstructions of vineyard crops [26]. To detect and position potted flowers, a ZED 2 stereo camera plus the YOLO V4-Tiny network were availed to obtain real-time 3D point cloud information related to flowers and establish an automatic detection model [27]. Jun et al. [28] combined 3D perception, manipulation, and an end effector to calculate the 3D coordinates of a targeted crop. They also performed the motion control of the manipulator of a tomato harvesting robot on the basis of 3D coordinates. According to the point matching method for the features of left and right images based on the cosine distance and vector modulus, binocular vision plus an optimized SIFT stereo matching algorithm were exploited to identify the contour and distance data of fruit trees [29]. Nevertheless, although the above crop recognition and detection methods based on traditional machine vision and stereo imaging are relatively easy to implement, the detection accuracy is not satisfying. Therefore, the approaches based on deep learning are needed to achieve the expected target.

In recent years, convolutional-neural-network-based (CNN) deep learning approaches have rapidly developed. Compared with conventional methods, CNNs extract different levels of features from input images, achieving accurate target detection through information classification and location regression [30,31]. The well-known CNN architectures, such as AlexNet, ResNet50, and VGG16, can be utilized for the classification and diagnosis of diseases as well as the quality inspection of fruits and vegetables [32,33,34,35]. Fu et al. [36] used an optimized GoogLeNet model to categorize apples, lemons, oranges, pomegranates, tomatoes, and colored peppers. They reduced the quantity of convolutional kernels of GoogLeNet and adjusted the structure of Inception, and these measures improved the accuracy of GoogLeNet by 2%. The above algorithms all belong to fundamental series of CNN, and state-of-the-art algorithms commonly utilize these models as the backbone network, and then perform different optimizations to form the one-stage [37] and two-stage detection methods [38]. The one-stage detection approaches include the SSD and YOLO series, which are used to perform uniform and dense sampling on the different positions of an image to generate bounding boxes of different scales and aspect ratios. After extracting features through the CNN, the candidate bounding boxes are directly classified and regressed. These methods have fast speed, but their level of accuracy is limited. The YOLOv3, YOLOv4, and YOLOv5 deep learning algorithms were applied for white grape yield estimation. Considering bunch occlusion, the YOLOv5x model for bunch number was able to estimate the amounts of bunches per plant with an average error of 13.3% per vine [39]. Initially, object detection was implemented through multimedia ontology infrastructure, and the semantic objects tested were video data in three different domains [40]. However, now, when the harvester performs target detection on asparagus or other crops, FCOS can be used for target detection in moving scenes [41,42,43]. For target detection, a cloud-side distributed framework for salient target detection based on an intelligent network can be further designed through a novel pyramid deep learning model; while retaining the local detail features of salient objects, it can effectively capture its global context features [44]. One-stage detectors such as RetinaNet can be applied to the visual recognition of harvesters, but in general, their accuracy cannot compete with two-stage detection approaches [45,46,47]. In the two-stage detection methods, the region proposal network is first used to obtain a series of sparse prediction boxes and then comprehensively input the feature map and prediction box into the fully connected layer to complete the classification and regression. Representative algorithms include Faster R-CNN and R-FCN, which are characterized by high precision but take a long time to process. The degree of ripeness and location of beef tomato fruits were acquired based on binocular imaging and R-CNN. The precision and the recall values of the mature fruits of this study were over 95% [48]. In [49], three deep learning models, YOLO-v3, CenterNet, and Faster RCNN, were compared in terms of their ability to detect vegetables and distinguish them from weeds. In the field of crop detection and recognition, crop image segmentation and extraction are crucial. A modified version of the Mask-RCNN algorithm added path aggregation and features to the optimized region extraction network and feature pyramid network, and had better performances in terms of precision, recall, AP, mAP, and F1-score values [50]. Mask RCNN can also be combined with the migration learning algorithm to detect cotton leaf diseases. The experimental results showed that the model’s accuracy is 94% [51]. Compared with the original Faster RCNN, the improved version enhanced the accuracy of object detection [52,53]. In terms of fitting target detection, the HRM-CenterNet, which combined the lightweight MobileNetV3 and CenterNet, had a smaller network dimension and faster speed than the original CenterNet [54]. In [55], 15 types of vegetables were utilized to measure the effect of the DNN-framework-based recognition system on each vegetable category; in addition, the performance of learning accuracy and loss for a vegetable recognition system based on Caffe and Chainer frameworks was evaluated, which showed that the results of Caffe were better than those of Chainer. Overall, the vegetable detection methods in smart agriculture need to attain a good balance between precision and speed.

## 3. The DA-MASK RCNN Model

**Figure 2:**
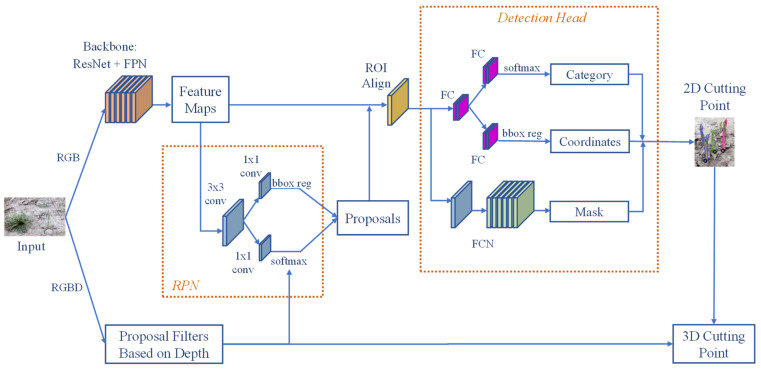
Structure of the DA-Mask RCNN model.

A two-stage object detection model usually consists of a backbone convolutional network, a region proposal network, and a detection head unit [56]. The function of a backbone network is to extract feature information from different levels from the input image. In this study, the detection head unit was made up of three modules, which were the category branch, coordinates branch, and mask branch. The category branch was responsible for classifying the green asparagus and background, while the coordinates branch obtained the exact location of the prediction box. The mask module was used to acquire more precise cutting points. The function of the region proposal network was to form candidate regions, predict the sample attributes (positive samples or negative samples) of the candidate regions, and preliminarily perform bounding box regression on the positive candidate regions. In the proposed DA-MASK RCNN model, a depth filter was added to the RPN unit, which is shown in Figure 2. In the proposed model, the depth information related to the corresponding RGB pixel was employed to acquire a more accurate binary classification of the anchor. Following the softmax function, the proposed depth filter re-checked the classified anchor to give the final decision. Although the added depth filter resulted in slightly longer processing time, it aided the softmax function in generating more accurate anchor classification.

### 3.1. Backbone Network

**Figure 3:**
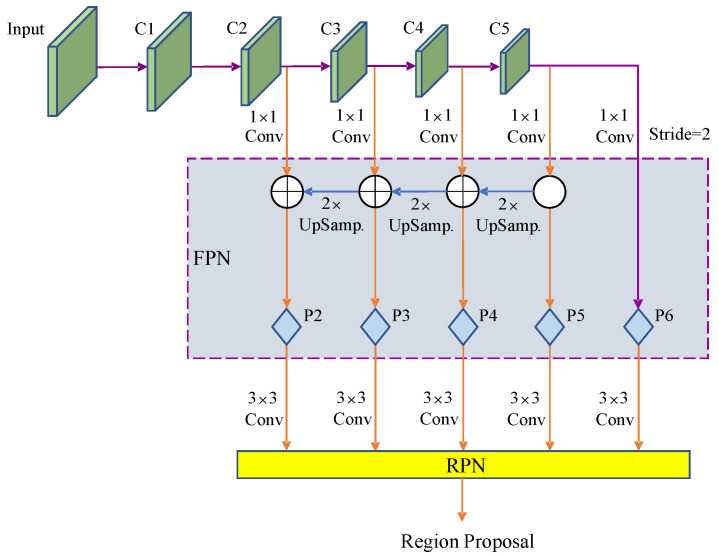
Structure of the backbone network.

In this study, a deep residual network (ResNet101) [57] and feature pyramid networks (FPNs) [58] were combined to form the backbone network. As shown in Figure 3, the left–right ResNet101 network obtained five-level feature maps C1, C2, C3, C4, and C5 from low to high. The low- and high-level feature maps contained more detailed information and semantic information, respectively [59]. The mapping of the convolutional features of the ResNet101 network is shown as follows:

(1) $$f_{k}^{l}=w_{k}^{l^{T}}\cdot e^{l}+b_{k}^{l}.$$

where *l* refers to the number of feature layers, *k* represents the number of convolution layers, $f_{k}^{l}$ shows the feature of $l^{th}$ layer after the convolution of $k^{th}$ layer, and $w_{k}^{l^{T}}$ means the weight of $k^{th}$ layer’s convolution; $e^{l}$ stands for feature of the $l^{th}$ layer, and $b_{k}^{l}$ is the convolutional bias of $k^{th}$ layer.

In some RPNs, the faster R-CNN and R-FCN models take the high-level feature map of the last stage of the feature extraction network as the input to the RPN, which improves the speed of model training and inference. However, high-level feature maps have low resolution, and therefore, they cannot effectively characterize objects of different scales, which means there are certain limitations, especially regarding the detection of small-sized objects. Lin et al. [60] used the multi-scale hierarchical structure contained in the CNN to design a feature pyramid network. By fusing the high-resolution features of shallow feature maps and the rich semantic data of deep feature maps, the detection accuracy of the deep inference model for multi-scale objects was improved, which is very appropriate for green asparagus detection.

In Figure 3, the feature maps of different stages from left to right, generated from the mixed depth-wise convolution, are shown. In stage *x*/*y* (*x* = 1, 2, ..., 5; *y* = 2, 4, ..., 32), *x* refers to the number of stages in which the feature map is located (C1–C5), and *y* represents the reduction factor of the feature map’s size at this stage. Stages 2–5 are input to the FPN unit after 1 × 1 convolution. The 1 × 1 convolution can ensure the consistency of channel numbers of the feature maps that are input to the FPN. The FPN unit upsamples the input high-level feature map in a right–left order to expand the resolution, and afterwards, the upsampled feature map will be fused with adjacent low-level features through addition. On one hand, the fused feature map is input to the subsequent network for prediction. On the other hand, it continues to be fused with the lower-layer feature map through upsampling. Mixed depth-wise convolution stages 2–5 correspond to the P2–P5 levels of FPN, and P6 is obtained by the downsampling in stage 5, which is used to generate prediction boxes in the RPN network and does not participate in the fusion operation. Each level in {P2, P3, P4, P5, P6} is responsible for processing the information of a single scale, corresponding to five-scale prediction boxes {16^2^, 32^2^, 64^2^, 128^2^, 256^2^}, each of which has three aspect ratios {1:1, 1:2, 2:1}, and a total of 15 prediction boxes are used to predict the target object and the background.

### 3.2. Improved Region Proposal Network Based on Depth Filter

Region proposal networks [61] are utilized to generate region proposals. An RPN sets multiple candidate anchors on the scale of an original image, and it is able to determine whether the anchors belong to the positive or negative class based on the softmax function. With the purpose of obtaining accurate proposals, it applies bounding box regression to correct the anchors. The positive anchors of the proposal layer of a classic RPN and the offset of related bounding box regression are applied to obtain proposals. In the field of smart agriculture, crops of all sizes need to be detected, and information related to depth can assist in determining proposals of small sizes.

**Figure 4:**
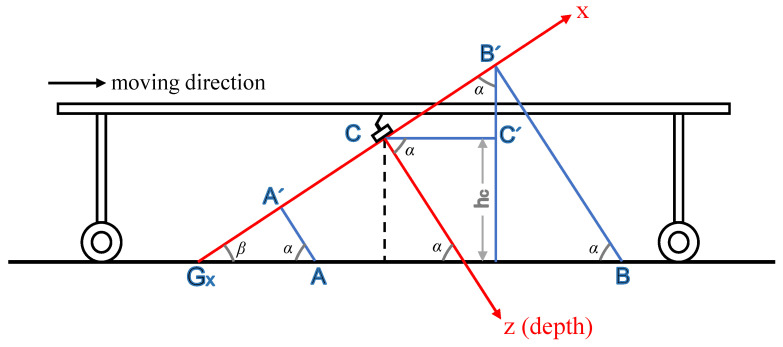
Sagittal illustration of the harvester and camera coordinate system.

As shown in Figure 4, in this study, the depth value corresponded to the *z*-coordinate value in the camera coordinate system. The camera was fixed under the harvester, and the y axis of the camera coordinate was perpendicular to the paper surface, namely the *xCz* coordinate surface. The moving direction of the asparagus harvester was parallel to the *xCz* surface. $h_{c}$ is the distance from the origin of the camera coordinate system to the ground, and α is the included acute angle between the ground and *z* coordinate. *β* is the complement angle of α. When the *x* coordinate value of the captured point is smaller than 0, the ground pixel can be determined using Equations (2) and (3), as follows:

(2) $$L_{A^{\prime }G_{x}}=\left|x_{g}\right|-\left|x_{A}\right|.$$

(3) $$\{\begin{array}{c} \mathrm{if}\frac{z_{A}}{L_{A^{\prime }G_{x}}}\in \left[\left(1-t_{f}\right)\tan\alpha ,\left(1+t_{f}\right)\tan\alpha \right],B\in P_{g}\\ \mathrm{otherwise},B\in P_{n} \end{array}.$$

where *L_A’Gx_* denotes the distance from point *A’* to point $G_{x}$, *x_g_* and $x_{A}$ represent the *x* coordinates of point $G_{x}$ and point *A* respectively, *P_g_* means the collection of ground pixels, *P_n_* refers to the collection of non-ground pixels, and *t_f_* stands for the tolerance factor.

When the *x* coordinate value of the captured point is greater than 0, the ground pixel is determined as follows:

(4) $$L_{B^{\prime }C^{\prime }}=x_{B}\cdot \cos\alpha .$$

(5) $$h_{B^{\prime }}=h_{c}+L_{B^{\prime }C^{\prime }}.$$

(6) $$\{\begin{array}{c} \mathrm{if}\frac{h_{B^{\prime }}}{z_{B}}\in \left[\left(1-t_{f}\right)\sin\alpha ,\left(1+t_{f}\right)\sin\alpha \right],A\in P_{g}\\ \mathrm{otherwise},A\in P_{n} \end{array}.$$

where $L_{B^{\prime }C^{\prime }}$ represents the distance between point *B’* and *C’*, $h_{B^{\prime }}$ means the distance between point *B’* and the ground, $x_{B}$ and $z_{B}$ are the *x* and *z* coordinate values of point *B* respectively.

In this study, the depth information related to the stereo camera was used to obtain a more accurate binary classification of the anchor. Originally, the classification was performed using the softmax function alone, and accordingly, the accuracy was very coarse. Following the softmax function, the proposed depth filter confirmed the positive anchor if the percentage of the ground pixel *R_p_* in the anchor was less than the threshold *t_d_*; otherwise, it would be revised to ‘negative’. If the negative anchor given by the softmax function had a higher ratio of ground pixel than the threshold, the final mark would be amended to ‘positive’; if not, the negative mark would be kept. The depth filter is depicted in Formula (7), where *L_i_* denotes the determined label of the input anchor. By utilizing the depth filter, we aimed to decrease the false positive number and false negative number, which were mainly caused by the analogy of loess pixels and asparagus pixels. The reduction in false positives and false negatives would result in higher precision and recall value, respectively.

(7) $$\{\begin{array}{c} R_{P}>t_{d},L_{i}=1\\ R_{P}\leq t_{d},L_{i}=0 \end{array}.$$

### 3.3. ROI Align

RoI Align is an approach used for the aggregation of regional features, and it effectively solves the inaccuracy problem which results from the two approximation operations of RoI Pooling in the Faster R-CNN. Meanwhile, it can improve the overall precision of a detection model. In this study, to avoid quantization errors, RoI Align adopted the bilinear interpolation algorithm. The pixel value corresponding to the sampling point was calculated using the pixel values of the four nearest points around the sampling point in each sub-region on the feature map. After this operation, the quantization error was avoided, and the performance on small targets was improved. In addition, the loss function defined by the Mask R-CNN was different from that of the Faster R-CNN.

**Figure 5:**
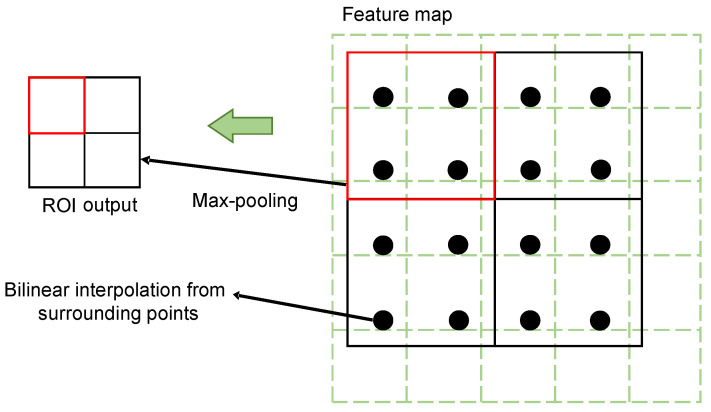
Illustration of ROI Align.

For each region of interest, the mask branch defined an *n* × *m* two-dimensional matrix, meaning that the detected region of interest directly corresponded to the branch of its class, which could be calculated directly. This avoided interclass competition and could effectively improve the classification performance. An illustration of ROI Align is shown in Figure 5.

### 3.4. Detection Head

The three-branch detection head consisted of category, coordinates, and mask modules. The category and coordinates branches were similar to those in the Faster RCNN model, which output the results of green asparagus determination and the position of the bounding rectangle. The FCN sub-model output the final mask of detected asparagus stalks. The FCN is an “end-to-end” image segmentation approach, in which the whole layers belong to convolutional layers. In addition, the FCN realizes the classification of image features at the pixel level, thereby realizing the semantic segmentation of images while preserving the spatial information of the original image. In this study, the output bounding rectangle of the coordinate branch may not have included the entire asparagus stalk, and therefore, the lowest point-based cutting point calculation would cause waste in the harvesting process. To solve this problem, we applied the FCN to obtain a more precise cutting point. For the asparagus mask, 20 pixels that were most adjacent to the ground pixels in the captured images were evaluated using Formulas (3) and (6) depicted in Section 3.2, where we used a stricter tolerance factor than determining the anchor class in the region proposal network. Afterwards, the mean 3D coordinate values of the 20 selected pixels were determined as the 3D cutting point.

### 3.5. Loss Function

The loss of the two-stage object detection framework is a multi-task concept, which is composed of an RPN loss and detection head loss. In addition, it can also be represented by the sum of classification loss, position regression loss, and mask loss. The classification loss utilizes the cross-entropy function [62] to acquire the error between the predicted category and the real category; the position regression loss uses the Smooth L1 function in order to calculate the position coordinate error between the ground truth and the prediction box and narrows the error range through multiple iterations, so that the ground truth and the prediction box have a large degree of overlap. Intersection over union (IoU) [63] represents the level of overlap between the prediction box and the ground truth box. It is an indicator of the measurement of accuracy of the prediction box in the domain of object detection. It can be backpropagated and optimized as an objective function. Some detection frameworks apply IoU to optimize the loss function to achieve better results, but when the prediction box does not overlap with the ground truth box, the values of IoU and the loss function are both 0. As there is no back propagation, learning cannot be performed. In view of the shortcomings of the IoU loss function, Zheng et al. [64] defines the DIoU loss function. DIoU correlates factors such as the distance, overlap rate, and scale between the ground truth and the prediction box, which effectively minimizes the length between two center points. Even if there is no overlap between the ground truth and the prediction box, a moving direction for the frame can be provided, making the position regression faster and more accurate. The DIoU loss function is specified as

(8) $$P_{diou}=\frac{\rho^{2}\left(b_{ctr}^{p},b_{ctr}^{gt}\right)}{d^{2}}.$$

(9) $$L_{diou}\left(b^{p},b^{gt}\right)=1-I_{iou}\left(b^{p},b^{gt}\right)+\frac{\rho^{2}\left(b_{ctr}^{p},b_{ctr}^{gt}\right)}{d^{2}}.$$

(10) $$I_{iou}\left(b^{p},b^{gt}\right)=\frac{b^{p}\cap b^{gt}}{b^{p}\cup b^{gt}}.$$

where $P_{diou}$ is the penalty term and $L_{diou}$ is the DIoU loss function; $b^{p}$ denotes the prediction box, and $b^{gt}$ means the ground truth box; d represents the diagonal length of the minimum bounding box of $b^{p}$ and $b^{gt}$; *ρ*(·) is the Euclidean distance function, $b_{ctr}^{p}$ refers to the origin of the prediction box coordinates, and $b_{ctr}^{gt}$ stands for the coordinates of the center point of the ground truth box; $I_{iou}\left(b^{p},b^{gt}\right)$ denotes the IoU between the ground truth box and the prediction box.

In this paper, we introduced DIoU as the loss function for position regression to construct the loss function of the proposed model. The overall loss function was composed of the loss function of RPN and detection head, each of which consisted of classification loss and position regression loss, while the detection head had an additional mask loss. The calculation of loss function is as follows:

(11) $$L_{t}=L_{r}\left(p_{l},m_{l}\right)+L_{h}\left(p,u,o,n\right).$$

(12) $$L_{r}\left(p_{l},m_{l}\right)=\frac{1}{N_{cls}}\sum_{l}L_{cls}\left(p_{l},p_{l}^{*}\right)+\delta \frac{1}{N_{reg}}\sum_{l}p_{l}^{*}L_{diou}\left(m_{l},n_{l}^{*}\right).$$

(13) $$L_{h}\left(p,u,o,n,s\right)=L_{cls}\left(p,u\right)+\delta \prime \left[u\geq 1\right]L_{diou}\left(o,n\right)+L_{m}\left(p,s\right).$$

where $L_{t}$ is the overall loss function of the proposed model, $L_{r}$ refers to the RPN network loss function, and $L_{h}$ stands for the loss function of detection head; l denotes the anchor box index, $p_{l}$ is the prediction probability of $l^{th}$ anchor box for two-class classification, and $p_{l}^{*}$ represents the discriminant value of the $l^{th}$ anchor box; $m_{l}$ refers to the prediction box corresponding to the $l^{th}$ anchor box, $n_{l}^{*}$ represents the ground truth box corresponding to the $l^{th}$ anchor box, and p denotes the predicted category probability; u means the label value of real category; δ,$\delta^{\prime }$ are the weight parameters; $L_{cls}$ represents the classification loss function; $N_{cls}$ refers to the number of sampled anchor boxes, and *N_reg_* means the number of positive and negative samples.

## 4. Test Setup for Asparagus Detection

### 4.1. Test Platform

In this study, the model training and cutting point positioning tests were carried out on two separate computers. The hardware of the computer used for model training is as follows: Intel Core (TM) i7-8550U was selected as the CPU model, the frequency of which was set as 3.80 GHZ; the memory of the computer was 32 GB, and we chose NVIDIA GeForce GTX MX150 as the graphics processing unit, the memory of which is 2GB. The image acquisition equipment is a Tuyang FM810 three-dimensional RGBD camera. The main hardware setup of the computer used in the green asparagus cutting experiment is given below: Intel Core (TM) i5-12500 was chosen as the CPU model, the frequency of which was set as 3.00 GHZ; in addition, the computer had 16 GB of memory, and NVIDIA GeForce GTX 1060 was chosen as the graphics processor, the video memory capacity of which is 6 GB. Regarding the software environment applied in the research project, we used the Windows 10 64 bit system as the OS, Python as the programming language (Python 3.6), PyCharm as the programming environment, and Keras as the deep learning framework.

### 4.2. Data Acquisition and Processing

**Figure 6:**
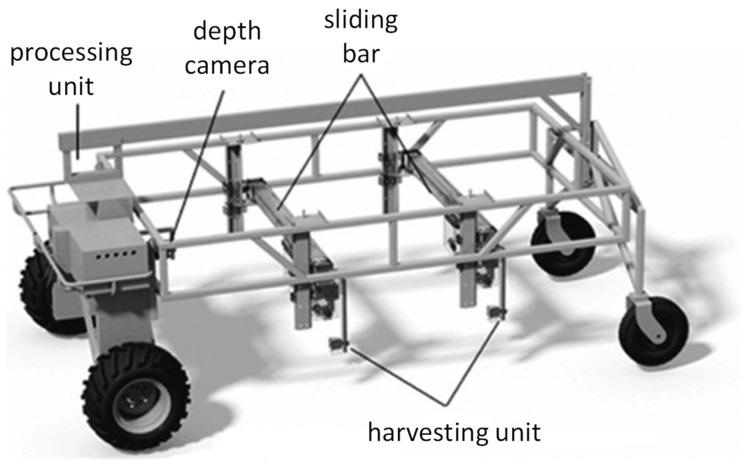
Illustration of the harvester.

**Figure 7:**
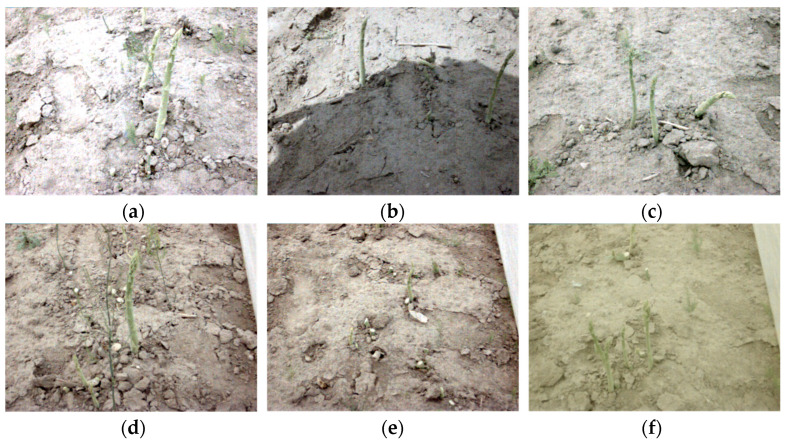
Samples of the acquired images of green asparagus: (**a**) Stalks in dataset S. (**b**) Stalks in dataset O. (**c**) Stalks in dataset C. (**d**) Single stalk in dataset D. (**e**) No harvestable stalk in dataset D. (**f**) Stalks in dataset N.

The test images were mainly collected in Shanghai, Suzhou, and Jiaxing, which are located in the basin of the Yangtze River in China. By capturing images of green asparagus in different regions, we could more effectively prove the robustness of the proposed model. To ensure the diversity of the data, images were captured under different weather and/or illumination conditions, namely ‘sunny’, ‘sunny but overshadowed’, ‘cloudy’, ‘greenhouse—daytime’, and ‘greenhouse—nighttime’. The positioning of the camera on the harvester is illustrated in Figure 6. A total of 13,500 images were obtained, and there are 3159, 2571, 2625, 2623, and 2522 images in datasets S, O, C, D, and N, respectively. The RGB and depth image dimensions were 640 pixels × 480 pixels. The Tuyang FM810 camera can capture images with the maximum dimension of 1280 × 960. However, since the mounting height of the camera is not high, the captured images of resolution of 640 × 480 contained sharp stalks of green asparagus. In addition, applying higher resolution would decrease the processing speed, which influences the real-time performance of the whole system. Samples of acquired images are shown in Figure 7. The acquired images were augmented by geometric transformation and color transformation, with the aim of enhancing the generalization performance of the training model. The augmented dataset was separated into a training set, validation set, and test set in the ratio of 8:1:1. These three datasets were independent and mutually exclusive. They were applied in the process of training, parameter optimization, and performance evaluation of the proposed model, respectively.

### 4.3. Model Training Strategy

In order to accelerate the convergence speed of the training process and improve the model’s performance, in this study, we adopted the stochastic gradient descent strategy to train the model alternately. The training parameters set the anchor point size to 32, 64, 128, 256, and 512. The number of ROIs for each image training process was 250, the initial learning rate was set as 0.001 and the momentum parameter of the learning step was 0.9. The training process was separated into three stages:

1. Train the backbone network and the three-branch network with the initial learning rate.
2. Train the overall network at 80 epochs with the initial learning rate and draw the epoch-loss-F1-score figure.
3. Select the appropriate epoch value through the figure.

### 4.4. Model Evaluation Metrics

To evaluate the proposed DA-Mask RCNN model objectively, the precision, recall, F1-score, and processing speed were selected as the evaluating metrics. In the experimental scenario of this research, green asparagus stalks were determined as positive samples, while other objects and backgrounds were treated as negative ones. The ratio between the number of green asparagus stalks correctly detected by the model and total number of green asparagus stalks predicted by the model was defined as the precision (*Pre*), which could be used to evaluate the model’s ability to identify positive samples. Similarly, the ratio between the number of correctly detected asparagus stalks and the actual number of positive samples was defined as the recall (*Rec*). The recall value could be used to quantify the ability to cover positive samples. The accuracy (*A_cc_*) indicated the overall performance in classifying the foreground and background, which was effective when the composition of the positive and negative samples was reasonable. The F1-score’s value relies on both precision and recall. The calculation of *Pre*, *Rec*, *F1-score*, and *A_cc_* are shown in Formulas (14)–(17).

(14) $$Pre=\frac{TP}{TP+FP}.$$

(15) $$Rec=\frac{TP}{TP+FN}.$$

(16) $$\text{F1-Score}=\frac{2Pre.Rec}{Pre+Rec}.$$

(17) $$A_{cc}=\frac{TP+TN}{TP+TN+FP+FN}.$$

where *TP* stands for true positive, and *FP* denotes false positive, *TN* denotes true negative, and *FN* represents false negative.

## 5. Experimental Results and Analysis

### 5.1. Experiments for Asparagus Detection

Table 1 shows the performance of the proposed model under different weather and illumination conditions, which are sunny (S), sunny but overshadowed (O), cloudy (C), greenhouse and daytime (D), as well as greenhouse and nighttime (N). The greenhouse and daytime had sufficient and stable illumination generated by both natural and artificial light sources, and therefore, it was the best scenario in this experiment, under which the precision, recall, and F1-score values were 0.993, 0.971, and 0.982, respectively. The precision value was 0.022 higher than that of recall, because the color components’ similarity to green asparagus and the loess in the captured images created more false negatives. The changing illumination and shade caused the deterioration of the feature, and hence, the performance of the proposed model in dataset O was worse than in other datasets with relatively more stable light conditions. Nevertheless, the recall value of 0.971 is sufficient for green asparagus detection tasks, which shows that the proposed depth filter is robust to the noises. The performance of the proposed model can compete with the classification approach using improved local ternary patterns and a multi-layer neural network [65]; however, there is no need to concretely design the feature extractor in the proposed DA-Mask RCNN model. The effectiveness of the model for datasets C and N was similar, and the precision values were 0.041 and 0.034 worse than the value of dataset D. Illumination deterioration, which created more false negatives, was the dominating factor affecting the decrease in the recall value. The averaged precision and recall values were 0.946 and 0.930, respectively.

### 5.2. Comparison with the State-of-the-Art Algorithms

A comparison between the proposed model and other models is established in this sub-section. Regarding the detection rate, the parameters of precision, recall, and F1-score were considered in the comparison. In addition, the processing speed of each model was measured by frame rate.

**Figure 8:**
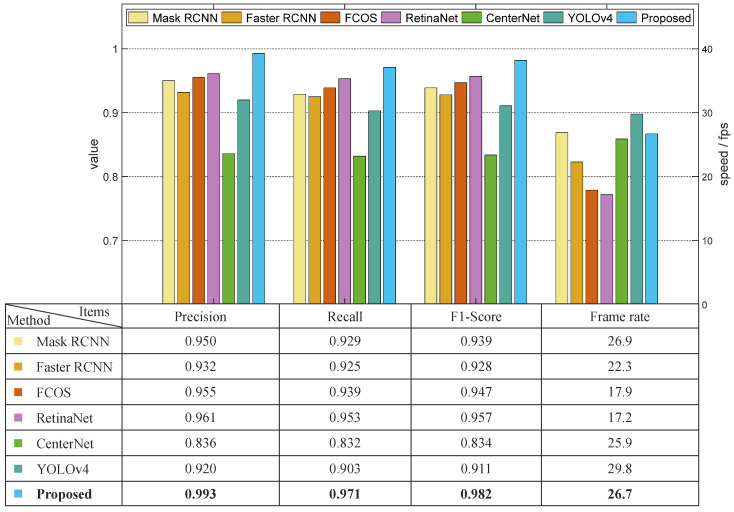
Results of comparison experiment using dataset D.

Datasets C, D, and N had relatively more stable illumination conditions, among which dataset D was chosen in the comparison experiment regarding stable light scenarios. The results are shown in Figure 8. The F1-score value of the proposed model was 0.042 higher than that of the Mask RCNN. The processing speed of the proposed model was slightly slower than that of the Mask RCNN model due to the calculation time needed for the depth filter. The precision, recall, and F1-score values of the proposed model were 0.061, 0.046, and 0.054 higher than those of the Faster RCNN, and the speed of the proposed model was 19.7% faster than that of the Faster RCNN. The Faster RCNN and the proposed model have some similarities in terms of network structure, however, the Faster RCNN uses VGG as its backbone network. Moreover, the Faster RCNN applies ROI pooling rather than ROI Align, the round off of which decreased the overall accuracy of the model. These two factors led to the less robust performance of the Faster RCNN compared with the proposed model. The average precision of FCOS and RetinaNet had good competitiveness compared with the proposed model, the values of which were only 0.038 and 0.032 less than our method, respectively. However, the frame rates of FCOS and RetinaNet were both less than 18.0 frames per second, which were much slower than the proposed model. YOLOv4 was shown to have the best processing speed (29.8 f/s), which was almost two times faster than FCOS and RetinaNet, but the one-stage structure meant that it had a relatively unsatisfying detection rate. Overall, the proposed solution was shown to improve upon the performance of the Mask RCNN model and outperformed other algorithms in terms of both accuracy and speed.

**Figure 9:**
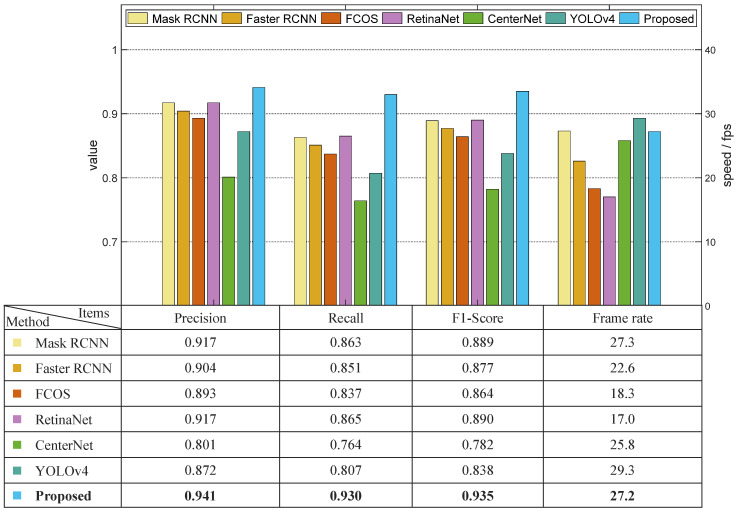
Results of the comparison experiment using dataset S.

The second comparison experiment was performed using dataset S to evaluate the effectiveness of different methods under natural sunlight. The results are shown in Figure 9. Similar to the previous experiment, the precision values of Mask RCNN, Faster RCNN, RetinaNet, and the proposed model were higher than 0.9, among which the proposed model attained the highest precision value (0.941). There is only one stage in the YOLOv4 model, the precision value of which was 0.872. CenterNet attained the lowest precision value of 0.801, which was 0.071 lower than that of YOLOv4. Compared with the results in the dataset regarding the daytime greenhouse conditions, the precision of each method decreased by a minimum of 0.028 and a maximum 0.062. Regarding the recall value, only the proposed model displayed a result higher than 0.9. Compared with the experiments using dataset D, the recall values of the Mask RCNN, Faster RCNN, FCOS, RetinaNet, CenterNet, and YOLOv4 using dataset S decreased by 0.066, 0.074, 0.102, 0.088, 0.068, and 0.096, respectively. The appearance of the green asparagus stalk and the loess were more similar under bright natural sunlight, which generated more false negatives, leading to the significant decrease in recall values. Only the proposed DA-MASK RCNN model’s decrease in recall value was smaller than 0.05. The depth filter in the proposed model increased the accuracy of the classification of the stalk and loess/background pixels in the RPN, and therefore outperformed other algorithms in this experiment, especially in terms of recall value. The frame rate hardly varied due to the consistency of the image dimension.

**Figure 10:**
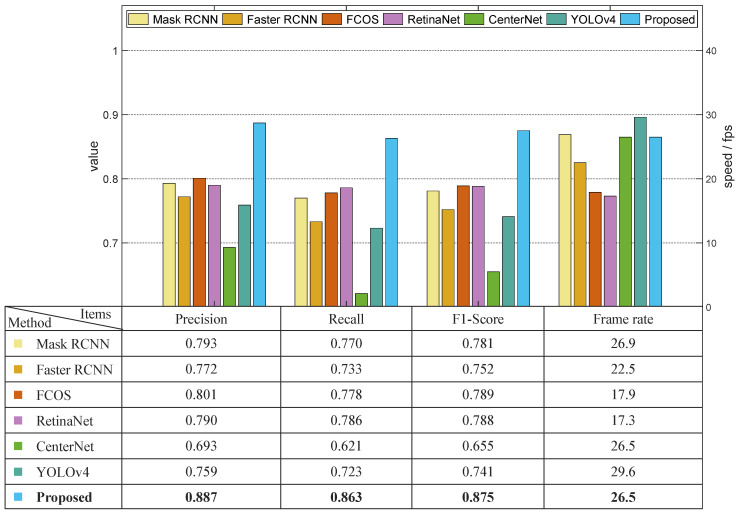
Results of comparison experiment using dataset O.

The third comparison experiment was conducted using dataset O, which means that the images were captured under natural sunlight, but the green asparagus stalks were cast in shadows. The results are illustrated in Figure 10. The precision value of CenterNet was only 0.693, with a recall value of 0.621. The Mask RCNN, Faster RCNN, RetinaNet, and YOLOv4’s precision values were 0.793 0.772, 0.790, and 0.759, respectively. The precision values of FCOS and the proposed DA-Mask RCNN model were higher than 0.8; they were 0.801 and 0.887, respectively. Compared with the experiments using images captured under sunlight without shadow (dataset S), the precision of these compared approaches decreased in the range of [0.054, 0.132], while the recall values of different methods decreased in the range of [0.067, 0.143]. The shadows deteriorated the features of green asparagus in the captured images, but it was shown that the proposed model could still reach an F1-score of 0.875, which again proves that the DA-MASK RCNN model outperformed the other methods in terms of vision-based green asparagus detection.

### 5.3. Ablation Experiments

**Figure 11:**
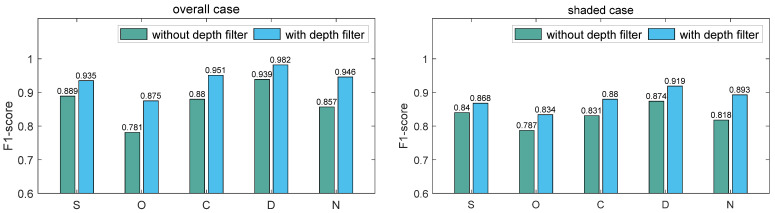
The results of ablation experiment for overall and shaded cases.

To more effectively verify the effectiveness of the proposed model, we conducted an ablation experiment regarding the depth filter. First, we built the model without the depth filter and performed the experiments using dataset S, O, C, D, and N. Second, we added the depth filter unit to assist the softmax function in anchor classification and performed the experiments on the same datasets. The results are shown in the overall case of Figure 11. For datasets D and S, the average F1-score values were improved by 0.043 and 0.046, respectively, after introducing the depth filter. More significant improvements corresponded to other weather and lighting conditions, including sunny but overshadowed, cloudy, as well as nighttime greenhouse, and the improvements in the average F1-score values were 0.094, 0.071, and 0.089, respectively. The added depth filter decreased the number of false positives caused by the loess pixel. Overall, the results of the ablation experiments prove that the depth filter unit contributed to the model’s overall performance.

Green asparagus stalks are more difficult to recognize when they are shaded by each other. Another ablation test using shaded stalks was performed to help verify the effectiveness of the proposed model. Using datasets S, O, C, D, and N, the original network attained average F1-score values of 0.840, 0.787, 0.831, 0.874, and 0.818, respectively. After applying the depth filter in the region proposal network, the average F1-score values reached 0.868, 0.834, 0.880, 0.919, and 0.893 for these five datasets, respectively. Improvements of 0.028, 0.047, 0.049, 0.045, and 0.075 were made in scenarios S, O, C, D, and N, respectively. The overall improvement in the detection capability was 0.049. Considering the existence of the shading effect, the application of the depth filter was shown to be very necessary.

### 5.4. Accuracy at Different Depths

**Figure 12:**
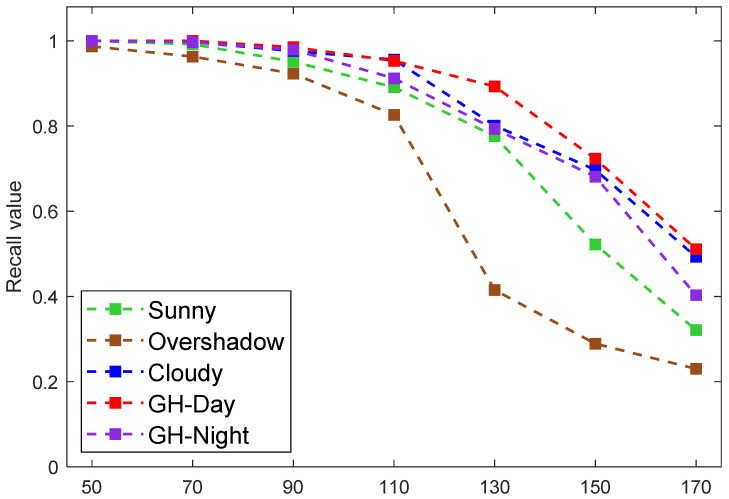
The average recall values at different depths.

This experiment assessed the model’s performance in recognizing green asparagus at various depths. The depth of the green asparagus stalk could vary from 50 cm to 150 cm depending on the position of the camera mounted on the harvesting machine and the camera angle adjustment. The experiment of precision-depth was conducted using datasets S, O, C, D, and N. We performed 50 measurements at different angles for each green asparagus stalk at the same depth. The test range of the depth was 50–170 cm, and the interval was 20 cm. The results are illustrated in Figure 12, in which the recall value was taken into account as the increase in depth caused more false negatives. When the depth data were below 90 cm, the average recall value was very ideal, being close to 100%. If the depth value was larger than 90 cm, the average recall value decreased gradually with the increase in the depth value. The results obtained using dataset O decreased more sharply than the others. When the depth reached 170 cm, the average recall value was only around 50%. Therefore, it is important to appropriately set up the camera on the harvesting machine.

### 5.5. Experiments for Asparagus Harvesting

Table 2 lists the results in terms of the detection rate and location rate of the cutting point in the harvesting experiments of green asparagus. The test images were captured under five different weather and illumination conditions, as in previous experiments. In Table 1, the values of one green asparagus stalk captured from different angles are shown to form a larger database. However, each asparagus stalk was captured only once in the cutting experiments, in which each dataset contained approximately 208 test asparagus stalks/images. The correct detection rate referred to the recall value as the harvesting blade executed the actual cutting operation.

**Figure 13:**
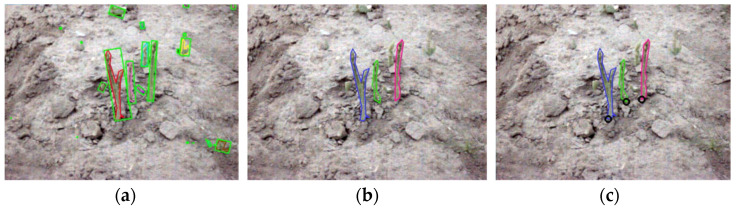
Illustration of the filtering in the cutting experiment: (**a**) All results. (**b**) Filtered results. (**c**) Cutting points of the asparagus.

Using dataset D, only two green asparagus stalks were missed in the detection process as a result of ideal illumination. The correct detection rate under condition D was 6.2%, 12.7%, 3.9%, and 5.5% higher than those of datasets S, O, C, and N, respectively. The relatively higher accuracy values in datasets C, D, and N were attributed to the stability of the light conditions. Among these three cases, the correct detection rate in dataset N was 1.6% and 5.5% lower than those of datasets C and D due to the lack of brightness and the completeness of chromatic light. For the two scenarios of ‘sunny’ and ‘sunny but overshadowed’, the changing natural illumination and/or shadow effect created unstable capturing conditions. The cutting operation was based on the 3D coordinates of the successfully detected asparagus stalks. The number of missed stalks in each dataset were two, zero, one, zero, and zero, respectively, which shows the robustness of the 3D reconstruction. As shown in Figure 13, the detection of small asparagus stalks was ignored in the cutting experiment to avoid waste.

### 5.6. Performance of the Proposed Model during the Training Process

**Figure 14:**
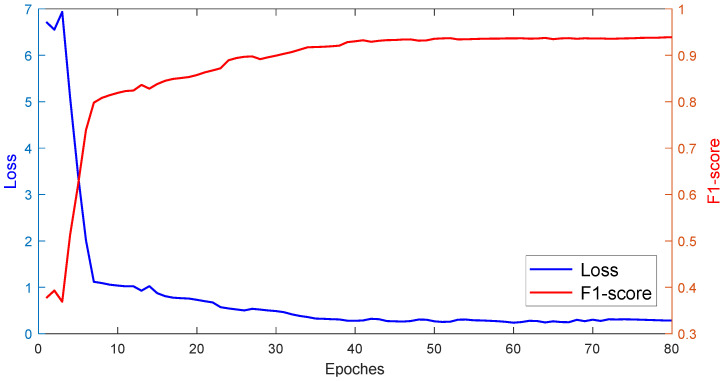
Loss and F1-score values at different epochs.

To more effectively demonstrate the performance of the proposed DA-Mask RCNN model in the training process, we present the F1-score value and the loss value of the model during the training process in Figure 14. In the initial phases of the training process, the precision value could still be very high, as precision only depends on the number of true positives and false positives. Furthermore, the recall value was very low in the initial phases. Therefore, we chose to use the F1-score to evaluate the overall performance of the model in training. The loss value converged in a very rapid manner to smaller values, and meanwhile, the F1-score increased from 0.377 to more than 0.8 in less than 10 epochs. Afterwards, the decreasing speed of the loss value and increasing speed of the F1-score value became slower from epoch 7. The two curves converged at around epoch 38, after which the values were almost stable. Hence, in this research, it was reasonable to choose the epoch value in the range of [40,50].

### 5.7. Discussion

In the comparison experiments under different weather and lighting conditions, the precision values of the proposed DA-Mask RCNN model and most compared algorithms were greater than those of recall values, owing to the fact that the color components’ similarity to the loess and green asparagus stalk in the RGB images generated more false negatives. Under natural sunlight scenarios, they had higher degrees of similarity, which led to a more significant decrease in recall values. Only the proposed DA-MASK RCNN model’s decrease in recall value was smaller than 5% under bright sunlight. The depth filter in the region proposal network increased the accuracy of the classification of the stalk and loess/background, and therefore, outperformed other algorithms, especially in terms of recall values. Under sunny but overshadowed scenarios, the shadows worsened the green asparagus’ features in the captured RGB images, but it was shown that the proposed model could still attain a robust performance sufficient for harvesting operation. The robustness under various weather and illumination conditions was a bonus caused by depth information, which can act as an important supplement in the detection procedure.

When there were some tiny asparagus stalks in the camera scene, two stage classifiers had a better performance than one-stage classifiers in terms of precision, recall, and F1-score values. The cause is that one-stage approaches are not robust to the objects of small sizes. In the depth-recall experiment, if the depth value was larger than 90 cm, the average recall values decreased gradually with the increase in the depth value due to the decrease in feature completeness. Meanwhile, in this test, the unsatisfying results in the scenarios of ‘sunny’ and ‘sunny but overshadowed’ were generated by the unstable capturing conditions and/or shadow effects. Moreover, the cutting operation of the blade was grounded on the 3D coordinates of the correctly detected asparagus stalks, in which there were almost no misses due to the robustness of the 3D reconstruction.

The processing speed of the proposed model was evaluated by frame rate. The DA-Mask RCNN model’s frame rate was slightly slower than that of the MASK RCNN algorithm, which was caused by the calculation time required by the depth filter. The speed of the proposed model was significantly faster than that of the Faster RCNN. The Faster RCNN and the proposed DA-Mask RCNN model have some similarities, e.g., network structure, but the Faster RCNN applies VGG as its backbone network; in addition, the Faster RCNN applies ROI pooling instead of ROI Align, whose round off resulted in the decrease in the overall accuracy of the model. Although the proposed model was not as fast as the compared one-stage models, it attained better balance between accuracy and speed.

## 6. Conclusions

In this article, we proposed a DA-MASK RCNN model for use in green asparagus detection based on the MASK RCNN and depth information. The appended depth filter assisted the softmax function in the process of anchor classification, which aimed to increase the classification capability of the RPN sub-net. The three-branch detection head obtained the category, position, as well as the mask of green asparagus stalks to acquire the cutting point. To verify the effectiveness of the proposed model, five datasets (S, O, C, D, and N) were built, and different types of experiments were designed and conducted. In the performance test using dataset D, the precision, recall, and F1-score values were 0.993, 0.971, and 0.982, respectively. The precision value for all the test images reached 0.942, which shows that the DA-Mask RCNN model is robust to the false positives caused by loess pixels captured under bright illumination. The accuracy of 3D reconstruction in the cutting experiments was 99.72%, meaning that this model can be applied to automatic green asparagus harvesting. Comparisons with other vegetable detection approaches were performed to demonstrate the superiority of the proposed DA-MASK RCNN model, especially under suboptimal illumination conditions. In the ablation test, the performance of the proposed model was compared with the model without a depth filter, and the results revealed that the improved architecture achieved better accuracy in terms of both single and shaded green asparagus stalks. In addition, the depth-precision experiments indicated that the best depth range for detection is from 70 cm to 90 cm. Overall, all of the tests validated the eligibility of the proposed DA-Mask RCNN in green asparagus harvesting.

In future work, we will conduct a follow-up model optimization to further improve the average performance using dataset N, which corresponded to the nighttime greenhouse scenario. The experiments will be conducted on other types of soil than loess. In addition, the application area of the proposed approach will be expanded to the harvesting process of other types of fruits and vegetables.

## Figures and Tables

**Table 1:** Performance of the proposed model under different weather and light conditions.

Weather and Light Conditions	Precision	Recall	F1-Score
Sunny	0.941	0.930	0.935
Sunny but overshadowed	0.887	0.863	0.875
Cloudy	0.952	0.950	0.951
Greenhouse—daytime	0.993	0.971	0.982
Greenhouse—nighttime	0.959	0.934	0.946
Average	0.946	0.930	0.938

**Table 2:** Results of cutting experiments.

Weather and Light Conditions	No. of Targets	No. of Correctly Detected Targets	No. of Failed Targets	Correct Detection Rate/%	No. of Correct Location	Correct Location Rate of Cutting Point/%
Sunny (S)	211	196	15	92.9	196	100.0
Sunny but overshadow (O)	206	178	28	86.4	178	100.0
Cloudy (C)	207	197	10	95.2	196	99.5
Greenhouse—daytime (D)	215	213	2	99.1	211	99.1
Greenhouse—nighttime (N)	203	190	13	93.6	190	100.0
Average	208.4	194.8	13.6	93.4	194.2	99.72

## Data Availability

Not applicable.
